# Effects of glucocorticoid replacement therapy in patients with pituitary disease: A new perspective for personalized replacement therapy

**DOI:** 10.1007/s11154-024-09898-6

**Published:** 2024-08-22

**Authors:** Sabrina Chiloiro, Alessandra Vicari, Ginevra Mongelli, Flavia Costanza, Antonella Giampietro, Pier Paolo Mattogno, Liverana Lauretti, Alessandro Olivi, Laura De Marinis, Francesco Doglietto, Antonio Bianchi, Alfredo Pontecorvi

**Affiliations:** 1 Dipartimento di Medicina e Chirurgia traslazionale, Università Cattolica del Sacro Cuore, Largo A. Gemelli, Rome, Italy; 2 Pituitary Unit, Department of Endocrinology and Diabetes, Fondazione Policlinico Universitario A. Gemelli, IRCCS, Rome, Italy; 3 Department of Neurosurgery, Fondazione Policlinico Universitario A. Gemelli IRCCS, Rome, Italy; 4 Department of Ageing, Neurosciences Head Neck, and Orthopedics Sciences, Università Cattolica del Sacro Cuore, Largo A. Gemelli, Rome, Italy

**Keywords:** Hypopituitarism, Diabetes insipidus, Pituitary adenoma, Pituitary neuroendocrine tumors, Pit-NET, Craniopharyngiomas, Hypophysitis, Histiocytosis, Germinoma, Metastasis

## Abstract

Secondary adrenal insufficiency (SAI) is an endocrine disorder due to impaired secretion of ACTH resulting from any disease affecting the pituitary gland. Glucocorticoid replacement therapy is mandatory to ensure patient survival, haemodynamic stability, and quality of life. In fact, a correct dose adjustement is mandatory due to the fact that inappropriately low doses expose patients to hypoadrenal crisis, while inappropriately high doses contribute to glucose metabolic and cardiovascular deterioration. This review analyses the current evidence from available publications on the epidemiology and aetiology of SAI and examines the association between glucocorticoid replacement therapy and glucometabolic and cardiovascular effects.

## Introduction

Central adrenal insufficiency (CAI) is an endocrine disorder resulting from an impaired secretion of the adrenocorticotropic hormone (ACTH), thus affecting the hypothalamic-pituitary-adrenal axis, because of any disease affecting the hypothalamus and pituitary gland or stalk. This form of adrenal insufficiency differs from primary adrenal insufficiency (PAI) due to adrenal disease, as aldosterone production is preserved and adequate in central adrenal insufficiency [1].

Symptoms and signs of adrenal insufficiency can be relatively non-specific and variable, progressing insidiously and making diagnosis challenging for clinicians. Nevertheless, its recognition and management cannot be ignored or delayed, as hypoadrenalism is a life-threatening condition and hormone replacement therapy with glucocorticoids is mandatory to ensure patient survival, hemodynamic stability, good clinical performance, and quality of life (QoL) [2–4].

Personalized doses of glucocorticoids in replacement therapy should be determined according to the clinical condition of the patient, in the absence of standardized biochemical markers, with the aim of mimicking the physiological circadian rhythm of cortisol secretion [5].

Some authors suggest adjusting glucocorticoid replacement therapy with some tools, such as measuring salivary cortisol [6] or adjust dose according to body surface area [7].

The risk of under- or overtreatment is real and quite common in clinical practice, even leading to serious hormonal imbalances, including electrolyte disturbances. Inappropriately low doses of glucocorticoids may expose patients to the risk of hypoadrenalism crisis, while inappropriately high doses of glucocorticoids may contribute to glucometabolic and cardiovascular changes in patients with AI, with overtime effects.

Increased cardiovascular risk and adverse metabolic profiles such as abdominal obesity, increased blood pressure, dyslipidaemia and glucose intolerance or impaired fasting glycaemia have been reported in patients with secondary adrenal insufficiency (SAI), affecting quality of life and long-term survival [8].

This review will analyse the current evidence from available publications on the epidemiology and aetiology of SAI. We also aim to examine the association between glucocorticoid replacement therapy, duration, and dose of treatment, and glucometabolic, cardiovascular, bone and immune effects.

## Methods and search strategy

The literature search was conducted in August 2023. We searched MEDLINE (PubMed database) with the data filter 2023–2013 using the following keywords: (ACTH deficiency OR central adrenal insufficiency OR secondary adrenal insufficiency OR pituitary hypoadrenalism OR secondary hypoadrenalism) AND glucocorticoid replacement therapy AND (pituitary tumor OR PitNET OR pituitary adenoma OR hypophysitis OR pituitary germinoma OR pituitary histiocytosis OR pituitary metastasis OR craniopharyngioma).

The articles met the following inclusion criteria: 1) written in English; 2) published between 1 January 2013, and 31 December 2023; 3) original studies and case series on the epidemiology, characteristics, and treatment of pituitary hypoadrenalism. Exclusion criteria included: articles written in languages other than English and single case report. We selected and analyzed relevant articles, focusing on information about the adrenal axis.

## Physiology of CRH-ACTH-cortisol axis

The hypothalamic-pituitary-adrenal axis (HPA) is part of the neuroendocrine system that plays a central role in human life [9]. The HPA is regulated by a negative feedback system, which is controlled by three systems: a delayed genomic-induced system (acting via transcriptional changes), an intermediate feedback system and a fast non-genomic system that appears to act at the cell membrane [10–12]. The complex crosstalk between the hypothalamic-pituitary-adrenal axis, the limbic system and sympathetic circuits is still under investigation. In flow chart 1 we summarize the concept schematically.

The hypothalamic-pituitary-adrenal (HPA) axis regulates the body's response to stress and various physiological processes. It orchestrates a complex hormonal cascade that regulates glucose, protein and lipid metabolism, hydro-electrolyte balance, cardiovascular function, immune system actions, and controls the ability to adapt to challenging situations, including cognitive behavior [5].

Cortisol acts on adipose tissue, increasing lipolysis in subcutaneous tissue of the extremities and activating lipogenesis in visceral and subcutaneous adipose tissue of the trunk. In addition, cortisol alters insulin action leading to hyperglycemia [13] and it increases proteolysis [14]. Glucocorticoids are also responsible for a crucial step in atherogenesis [15]. Finally, cortisol is associated with cognition, particularly visual memory, and learning [16].

The rate of cortisol production in a healthy organism is around 20 mg/day, with a well-defined circadian rhythm regulated by the CLOCK (Clock Circadian Regulator) and BMAL1 (Basic Helix-Loop-Helix ARNT Like 1) genes [17, 18].

## Pathology of CRH-ACTH-cortisol secretion

Adrenal insufficiency is the clinical manifestation of deficient glucocorticoid production or activity. It may result from primary adrenal insufficiency or from a disorder of the hypothalamic-pituitary axis, secondary or tertial adrenal insufficiency (respectively SAI and TAI) [19].

Primary Adrenal Insufficiency (PAI), or Addison's disease, is defined as the clinical manifestation of glucocorticoid and/or mineralocorticoid deficiency due to failure of the adrenal cortex. The most common causes of PAI are autoimmune, infectious, neoplastic, and genetic disorders, but iatrogenic conditions are also emerging as a possible etiology [20]. Otherwise, central adrenal insufficiency is defined as glucocorticoid deficiency due to ACTH deficiency in pituitary disease leading to SAI or impaired hypothalamic function with inadequate CRH production, usually due to exogenous administration of glucocorticoids (TAI) [21, 22].

The prevalence of PAI is estimated to be 82–144 cases per million population, while the prevalence of SAI is around 150–280 per million population. Data on mortality are conflicting, but increased mortality has been consistently reported in young patients, infections and/or acute adrenal insufficiency [23].

The onset of adrenal insufficiency may be gradual. The symptoms and signs of adrenal insufficiency depend on the rate and extent of loss of adrenal function: severe fatigue, anorexia, orthostatic hypotension, nausea, vomiting, abdominal pain, muscle, and joint pain are characteristic of this condition. Patients also commonly develop electrolyte abnormalities such as hyponatremia, hyperkalemia, and hypoglycemia. Skin hyperpigmentation and salt craving are common in patients with PAI, but not in patients suffering from SAI [2, 24]. In addition, most studies have found reduced quality of life and psychological morbidity compared to the general population. The mechanisms involved are still unclear, but it is likely that the lack of a physiological circadian rhythm of the hypothalamic-pituitary axis and other concomitant pituitary hormones deficiencies play a relevant role [25].

CAI has been described in the context of many hypothalamic and pituitary disorders, including pituitary adenomas, craniopharyngiomas, hypophysitis, histiocytosis, germinomas and metastases.

The diagnosis of hypoadrenalism remains challenging, because of the variability of clinical tests and the inconsistency of laboratory parameters. Basal cortisol levels may suggest the diagnosis of adrenal insufficiency, but if the basal cortisol level is inconclusive or the clinical assessment is inconsistent with cortisol levels, an ACTH stimulation test is required.

Glucocorticoid replacement therapy is therefore a necessary life-saving treatment for patients with adrenal insufficiency of any etiology.

It should be emphasized that in patients with multiple pituitary deficiencies, hormone replacement therapy for hypocortisolism must be given first to avoid a hypoadrenalism crisis [26]. Replacement of other pituitary axes, especially the thyroid axis, may slatentize a misdiagnosed hypoadrenalism. The hypoadrenalism or Addisonian crisis is a life-threatening situation and the most feared endocrine emergency [27]. The incidence is 5–10 cases per 100 patient-years and the mortality rate is 0.5/100 patient-years [28]. It is characterised by a variety of symptoms and signs such as diarrhoea, nausea, vomiting, malaise, dizziness, low blood pressure which rapidly progresses to hypovolemic shock characterised by hypotension, tachycardia and cerebral hypoperfusion. Laboratory tests usually show hypoglycaemia, hyponatraemia, hyperkalaemia, acute kidney injury due to hypoperfusion.

Nowadays, the pharmacological armamentarium for the treatment of hypoadrenalism has been enriched with a variety of compounds. Table 1 summarizes the different therapeutic approaches [6, 29].

**Table 1:** Different therapeutic approaches in glucocorticoid replacement therapy: formulations and usage aspects

	Different therapeutic approaches in glucocorticoid replacement therapy						
	Short acting glucocorticoids [6, 29]		Sustained-release glucocorticoids		Long-acting glucocorticoids		Continuous subcutaneous hydrocortisone
	Hydrocortisone	Cortisone acetate	Plenadren^®^	Chronocort^®^	Prednisone	Dexamethasone	
Positive aspects	• safety, • low cost		• Mimics physiological secretion rate more accurately [30, 31], • benefits on immune system, • reduced risk of osteopenia/osteoporosis [32], • benefits on lipid and glucose metabolism [33]	• benefits on immune system, • reduced risk of osteopenia/osteoporosis [32], • benefits on lipid and glucose metabolism[33], • better mimics the circadian pattern [35] • mimics nocturnal cortisol rise [7]	• more useful in primary adrenal insufficiency and congenital adrenal hyperplasia		• Mimic physiological secretion rate [39, 40]
Negative aspects	• No reproduction of physiological secretion rate		• not always effective on fatigue [34], • does not mimic nocturnal cortisol rise [35], • no reproduction of pulsatile secretion pattern [36]	• no reproduction of pulsatile secretion pattern [36]	• No reproduction of physiological secretion rate, • no recovery of spontaneous ACTH secretion [2]		• discomfort [37]

Short-acting glucocorticoids (hydrocortisone and cortisone acetate) are the most used therapy, both because of their safety and because of the cost of slow-release analogues. Hydrocortisone is the already active cortisol analogue, whereas cortisone acetate requires hepatic activation by the 11b-HSD1 enzyme. In general, the total daily dose (which is approximately 15–25 mg/die for hydrocortisone and 20–35 mg/die for cortisone acetate) is divided into two or three doses, according to patient’s age, with the aim of reproducing the endogenous production rhythm and supporting the patient throughout the day.

Sustained-release glucocorticoids seem to mimic the physiological secretion more closely, avoiding the peaks of traditional formulations [30, 31]. They also appear to be associated with benefits in the immune system, bone [32] and lipid and glucose metabolism, particularly in patients with PAI [33]. Nevertheless, it has been observed that the clinical impact of dual-release hydrocortisone is not always evident, particularly in relation to fatigue [34]. Moreover, it has been shown that Plenadren^®^ [35] is not able to mimic nocturnal cortisol rise, whereas Chronocort^®^ better mimics the circadian pattern of cortisol secretion and simulates the overnight increase in cortisol release [7], but still lacks the pulsatile secretion pattern [36].

Long-acting glucocorticoids (prednisone and dexamethasone) are less preferred in the treatment of patients with secondary hypoadrenalism, as there is a high risk of cushingoid side-effects and suppression of the HPA, particularly in patients with partial ACTH deficit, as recently reported by Husebye et al. [37].

Finally, studies with continuous subcutaneous hydrocortisone infusion have been performed, but the results have not shown any advantages over other formulations, with increased patient discomfort [38]. Nevertheless, the advent of nanopumps and the reduction in the size of reservoirs have facilitated the emergence of novel perspectives [39], such as a better reproducing of physiological secretion rate [40]: new pharmacokinetic studies are ongoing [41]. Table 2 summarizes the different strengths and functions of each glucocorticoid.

**Table 2:** different power and function of each glucocorticoid

Molecule	equivalent dose	Anti-inflammatory power	Mineralocorticoid power	Half-life
Hydrocortisone	4,1	1	1	< 12h
Cortisone acetate	5,2	0,8	0,8	< 12h
Prednisone	1	0,8	0,8	12-36h
Triamcinolone	0,8	4	0	12-36h
Dexamethasone	0,15	4	0	> 48h
Methylprednisolone	0,8	25	0	12-36h
Fluprednisolone	0,31	15	0	12-36h
Betamethasone	0,41	10	0	> 48h
Deflazacort	1,16	4,5	0,25	> 12h

Glucocorticoid replacement therapy lacks gold standard monitoring parameters and is also burdened by numerous inter- and intra-individual variations, making its management extremely complex. Clinical assessment is still the most reliable parameter for assessing the efficacy of glucocorticoid replacement therapy. In the absence of markers to determine the appropriate dose, patients often experience multiple side effects because of over- or under-treatment.

In the former case, weight gain, insomnia and peripheral oedema are usually described, whereas in the latter case patients report nausea, loss of appetite, anorexia, fatigue, and hyperpigmentation (the latter only in primary hypoadrenalism). Cardiovascular side effects, metabolic abnormalities (hyperglycemia, dyslipidemia, abdominal obesity), osteoporosis, reduced quality of life and increased mortality are the main long-term problems of over-replacement [29, 42].

Bornstein et al. suggested tailoring therapy to the patient's lifestyle by asking about habits, work patterns, energy, concentration, and daytime sleepiness, and highlighted that monitoring serum or salivary cortisol diurnal curves may be useful, especially if malabsorption is suspected, whereas ACTH levels usually lead to over-replacement [43]. Andela et al. pointed out that QoL in patients with primary and secondary adrenal insufficiency correlates more with hydrocortisone dosage than with long-term systemic cortisol levels. In this study, they proposed the measurement of hair cortisol levels (CORThair) as a parameter to assess chronic systemic cortisol exposure [44]. Pazderska et al. emphasized that patient education and an accurate history and clinical examination are fundamental, as serum cortisol and ACTH and 24-h urinary free cortisol excretion do not accurately reflect tissue exposure to cortisol under glucocorticoid replacement [45]. A recently introduced approach is the use of epigenetics in the long-term evaluation of exposure to cortisone. DNA methylation in NR3C1, SLC6A4 and KITLG genes resulted in HPA axis dysregulation in non-tumoral hypercortisolism [46–49]. Other studies have been conducted on gene expression and its changes following the transition to dual-release hydrocortisone [50].

Despite the available studies, correct personalization of glucocorticoid replacement therapy is difficult to achieve, and tailored therapy remains a hot topic of debate.

## Epidemiology of hypoadrenalism in different pituitary pathologies

CAI has been described in the context of many hypothalamic and pituitary disorders. Central hypoadrenalism has a relevant incidence in several pituitary diseases. Table 3 summarizes the most frequent causes of hypopituitarism and SAI [6, 51–53], including pituitary adenomas/neuroendocrine tumors, craniopharyngiomas, hypophysitis, histiocytosis, germinomas, teratoma and metastases.

**Table 3:** Differential causes of hypopituitarism and SAI

Congenital - Genetic - Acquired
Neoplastic - Pit-NET (functioning and non-functioning) - Craniopharingioma - Meningioma - Cysts - Germinoma - Glioma - Astrocytoma - Ganglioneuroma - Teratoma - Chordoma/Chondrosarcoma - Pituicytoma - Ependymoma - Pituitary carcinoma - Metastases
Infiltrative - Neurosarcoidosis - Langerhans cell histiocytosis - Hemochromatosis
Inflammatory / immunological - Hyphophysitis - Granulomatous
Infectious - Bacterial - Fungal - Parasites - Tuberculosis - Syphilis Traumatic / Vascular - Pituitay apoplexy - Traumatic brain injury - Sheehan’s syndrome - Intrasellar carotid artery - Aneurysm - Subarachnoid haemorrhage
Empty sella
Iatrogenic / Medications - Surgery - Radiotherapy - Opiates - Glucocorticoids - Megestrol acetate - Somatostatin analogues - Immuno-checkpoint inhibitors
Idiopathic

### Pituitary tumors

Pituitary adenomas, namely pituitary neuroendocrine tumours (PitNETs), are the most common neoplasms of the pituitary gland. PitNETs are incidental findings in up to 20% of autopsies [54]. Pituitary tumors are traditionally classified as small PitNETs (< 10 mm), large PitNETs (> 10 mm) and giant PitNETs (> 40 mm). Pituitary carcinomas are very rare (0.1–0.2%) [55], but the uncertain biological behavior of these tumours poses a challenge to clinicians [56, 57]. Two thirds may secrete excess hormones: secreted hormones often include prolactin (32%-51%), growth hormone (9%-11%) or adrenocorticotropic hormone (3%-6%). Thyroid-stimulating hormone-secreting and gonadotropin-secreting tumours are rare (< 1%) [58].

Anterior hypopituitarism is present in approximately 50% of patients at the time of PitNET diagnosis and is more common in larger (mainly non-secreting) PitNETs, in males and in older individuals [59].

The frequency of SAI ranges from 8 to 55% of patients affected by PitNETs, mainly in association with other pituitary hormone deficits, such as TSH deficiency (5%-42.5%), FSH/LH deficiency (4–62.4%), GH deficiency (2.3–60%), as shown in Table 4 ([59–63]).

**Table 4:** Epidemiology of ACTH deficit and other pituitary hormones deficit in pituitary adenoma/neuroendocrine tumors

**Authors (Reference)**	**Num of patients**	**Age at diagnosis** **mean (ranges)**	**Gender** **M/F (%)**	**Timing of pituitary function evaluation**	**Size**	**Frequency of pituitary hormones deficit**			
						**ACTH-deficit**	**TSH-deficit**	**FSH/LH-deficit**	**GH-deficit**
Grayson et al. [60]	89/279^a^	54 yrs	M: 52% F: 48%	N.A.	9.6% < 10 mm, 90.4% 10–40 mm	14.8%	14.8%	6.8%	2.3%
Grayson et al. [60]	190/279^b^			N.A.	14.7% < 10 mm, 85.3% 10–40 mm	8.6%	4.8%	4.3%	0%
Mukai et al. [61]	63	58 yrs (47–70)	M: 66.7% F: 33.3%	After pituitary surgery	Median diameter 2.56 (2.06–3.16) cm	55%	30%	41%	59%
Mavromati et al. [62]	137	59 yrs (28–86)	M:59.1% F: 40.9%	Before pituitary surgery	Median diameter 2.48 (1–5) cm	30.8%	41%	62.4%	29.9%
				After pituitary surgery		27.4%	29.3%	40.8%	17%
Hwang et al. [63]	209	58 yrs (49–66)	M: 56.5% F: 43.5%	Before pituitary surgery	Tumor volume 4.9 (2.9–10.1) cm^3^ Median diameter 2.6 (2–3.1) cm	42.5%	42.5%	76.8% only males	25.8%
				After pituitary surgery		9.7%	24.3%	15.4% only males	19.4%
Jahangiri et al. [59]	305	57 (16–93)	M: 55.7% F: 44.3%	Before pituitary surgery	Median diameter 2.2 (3–7.1) cm	13%	26%	20% males 16% females	19%

*N.A.* data not available, *M* male, *F* female, *ACTH* AdrenoCorticoTropic Hormone, *TSH* Thyroid-Stimulating Hormone, *FSH* Follicle-Stimulating Hormone, *LH* Luteinizing Hormone, *Yrs* years

^a^Data on 89 patients were collected before evaluation from a multidisciplinary team care

^b^Data on 190 patients were collected after evaluation from a multidisciplinary team care

Some authors have investigated the effect of surgical removal of PitNET on pituitary function. Masui et al. reported the occurrence of new pituitary hormone deficiency after surgery in 10–30% of a cohort of 63 patients [64]. Mavromati et al. [62] and Hwang et al. [63] described a clinically significant recovery of pituitary function in their cohort of 209 and 305 PitNETs, respectively, ranging from 3.4% to 32.8% for ACTH-D, 11.7% to 18% for TSH-D, 21.6% to 61.4% for FSH/LH-D in males, 0.4% to 12.9% for GH-D. Mavromati et al. (who underwent a hormonal work-up up to 6 months before surgery, assessing central adrenal insufficiency by basal plasma cortisol levels and a standard 250 µg ACTH stimulation test) found that male patients and patients with hyperprolactinemia at diagnosis were more likely to experience recovery of pituitary function after surgery. In this case, the percentage of hypoadrenalism was 30.8% before surgery and 27.4% after surgery [62]. Hwang et al. reported an encouraging result with 42.5% of patients suffering from central hypoadrenalism before surgery and 9.7% after surgery. Diagnosis was made with the 8 am plasma cortisol and 250 mcg ACTH test in patients with a basal level < 15mcg/dL. In the same casuistry, the incidence of GHD was reported to be 25.8% before surgery and 19.4% after surgery, showing a less significant improvement when compared to adrenal restoration [63].

A multidisciplinary team and the management of these patients in a center of reference for pituitary disease may improve the quality of care and improve the surgery safety and efficacy [60].

### Craniopharyngiomas

Craniopharyngiomas originate from the epithelium of Rathke's pouch, a vestigial remnant of the oral structures. It is the most common sellar tumour in children [65, 66]. The histotypes reported to date are adamantinomatous and papillary, the latter being described mainly in adults. Signs and symptoms at presentation vary according to the age of the patient at diagnosis and the location of the neoplasm (intrasellar or suprasellar) and are mainly short stature, precocious puberty, and polyuria. Headache (45.5%), visual impairment (39.5%) and nausea (33.0%) were the most common symptoms reported in a cohort of 200 childhood-onset craniopharyngiomas [67]. Pituitary hormone deficiency at diagnosis affects between 60 and 90% of patients, with similar frequencies in childhood-onset and adult-onset disease [68], as summarized in Table 5 ([67, 69–74]). Abalı et al. reported secondary hypoadrenalism in 27 of 29 (93.1%) patients with childhood-onset craniopharyngioma [69]. They also reported the presence of other pituitary deficits: secondary hypothyroidism in 28 patients (96.5%), precocious puberty in 10 patients (34.5%), hypogonadotropic hypogonadism in 15 patients (51.7%), and AVP-D in 26 patients (89.6%). Tosta-Hernandez et al. [71] reported an incidence of secondary hypoadrenalism, in 52/57 patients during follow-up and described overweight in this pediatric population. In the single-centre cohort study by Wijnen et al. [75] pituitary hormone deficiency (98%), visual disturbances (75%) and obesity (56%) were the most common long-term health conditions. They also pointed out that childhood onset was associated with an increased incidence of all long-term problems compared with adult onset. In another study [74], the same authors highlighted that people with craniopharyngiomas are at higher risk of developing metabolic syndrome, in which case a prompt treatment is strongly recommended to reduce long-term complications and ensure a good quality of life [76, 77]. Jazbinšek et al. reported that the prevalence of at least one affected pituitary axis (diagnosed using data collected from medical records at time of diagnosis) increased from 54% at presentation to 100% at follow-up in childhood-onset craniopharyngiomas and from 41 to 93% in adult-onset craniopharyngiomas. In addition, they highlighted that the childhood-onset adamantinomatous subtype was more frequently associated with GH deficiency, AVP-D, or panhypopituitarism. Finally, they observed that metabolic syndrome was diagnosed in 80% of childhood-onset and 68% of adult-onset patients at follow-up [70].

**Table 5:** Epidemiology of ACTH deficit and other pituitary hormones deficit in craniopharyngiomas

**Authors (Reference)**	**Num of patients**	**Age at diagnosis** **mean (ranges)**	**Gender** **M/F (%)**	**Timing of pituitary** **function evaluation**	**Frequency of pituitary hormones deficit**				
					**ACTH-** **deficit**	**TSH-** **deficit**	**FSH/LH-** **deficit**	**GH-** **deficit**	**AVP-** **deficit**
Abalı et al. [69]	29	8.7 (0.7–16.9)	M: 62% F: 38%	N.A.	93.1%	96.5%	51.7%	N.A.	89.6%
Sončka Jazbinšek et al. [70]	46	19 childhood-onset (< 18 yrs, mean age 10,6)	N.A.	N.A.	94.7%	94.7%	84.2%	94.7%	78.9%
		27 adult-onset (> 18, mean age 52)	N.A.	N.A.	74.1%	81.5%	70.4%	51.8%	70.4%
Cavalcanti Tosta-Hernandez et al. [71]	57	9.6	M: 61.4% F: 38.8%	N.A.	91.2%	93%	52.6%	96.5%	80.7%
Miao et al. [67]	200	8.1	M: 65% F: 35%	Before surgery	28.8%	16.3%	N.A.	N.A.	11.8%
				After surgery	56.2%	70.3%	N.A.	N.A.	85.5%
Capatina et al. [72]	107	35 childhood-onset	M: 45.7% F: 54.3%	N.A.	82.8%	85.7%	N.A.	74.2%	68.2%
		72 adult-onset	M: 61.1% F: 38.9%	N.A.	75%	75%	N.A.	73.6%	34.3%
Van Santen et al. [73]	112	25 yrs	M: 51% F: 49%	N.A.	85%	96%	91%	94%	68%
Wijnen et al. [74]	178	47 yrs	M: 49.4% F: 50.6%	N.A.	81.5%	91.6%	87.1%	83.1%	62.3%

*N.A.* data not available, *M* male, *F* female, *ACTH* AdrenoCorticoTropic Hormone, *TSH* Thyroid-Stimulating Hormone, *FSH* Follicle-Stimulating Hormone, *LH* Luteinizing Hormone, *Yrs* years, *AVP* Arginine VasoPressin, *GH* Growth Hormone

### Hypophysitis

Hypophysitis is an inflammatory disease of the pituitary gland, classified as primary or secondary depending on the etiology. The primary forms are known to have an autoimmune etiology, whereas the secondary forms are triggered by a specific etiology such as sellar and parasellar lesions, systemic autoimmune diseases, infectious diseases or drugs, mainly immune checkpoint inhibitors [78].

Lymphocytic/autoimmune hypophysitis is typically associated with pregnancy and the puerperium and should be distinguished from Sheehan's syndrome [79].

Hypophysitis is characterized by symptoms related to pituitary hormone deficiency, headache and visual disturbances associated with the mass effect of an enlarged pituitary gland and infundibulum. Approximately 50% of patients with primary hypophysitis suffer for headaches, while 10% to 30% of patients have visual disturbances related to mass effect.

Patients with hypophysitis typically have some degree of anterior and/or posterior pituitary dysfunction [80, 81], due to inflammatory involvement of the pituitary and/or stalk [82–84]. According to Amereller et al., female sex predominates in hypophysitis, and the most common symptoms are fatigue (52%), headache (38%) and polyuria/polydipsia (38%). In addition, 42% of patients have concomitant autoimmune disease. Hypopituitarism is a clinically relevant condition in patients with primary autoimmune hypophysitis (PAH). The occurrence of ACTH deficiency (all diagnosed by 8 a.m. and/or random plasma cortisol and ACTH and ACTH test when necessary) is described in 22–75% of reported case series, TSH deficiency in 14%-75% of cases, FSH-LH deficiency in 31–75%, GH deficiency in 9–62% and AVP-D in 17–82% ([80, 81, 85–93]), with variable prevalence in patients with adeno-hypophysitis, infundibulo-neuro-hypophysitis or pan-hypophysitis [94].

Over the past two decades, immune checkpoint inhibitors (ICIs) have been approved for the treatment of several types of cancer and have represented a breakthrough in the field of cancer immunotherapy. Treatment with ICIs has been associated with a high incidence of immune-related side effects, including those affecting the endocrine system, particularly the thyroid and pituitary glands [94–96]. As the use of ICIs increases, the likelihood that oncologists will be confronted with this side effect is likely to increase, as is the diagnosis of immunotherapy induced hypophysitis (IIH). ACTH deficiency is the most common form of pituitary dysfunction in patients treated with ICIs. In this clinical setting, ACTH deficiency may be isolated or associated with reduced or absent secretion of other pituitary hormones. As shown in Table 6 [97], TSH and FSH/LH deficiencies are most associated with ACTH deficiency in patients with ICI-induced pituitary dysfunction, ranging from 35 to 100% of cases and 38% to 82% of cases, respectively [106–109]. The diagnosis of SAI was made by measuring plasma ACTH and cortisol, but without an ACTH test.

**Table 6:** Epidemiology of ACTH deficit and other pituitary hormones deficit in primary and secondary hypophysitis

**Authors (Reference)**	**Etiology**	**Num of patients**	**Age at diagnosis** **mean (ranges)**	**Gender** **M/F (%)**	**Frequency of pituitary hormones deficit**				
					**ACTH-** **deficit**	**TSH-** **deficit**	**FSH/LH-** **deficit**	**GH-** **deficit**	**AVP-** **deficit**
Langlois et al. [80]	PAH	3	57 yrs (30–73)	M: 33.3% F: 66.7%	33.3%	N.A.	N.A.	N.A.	66.7%
Angelousi et al. [85]	PAH	22	41.9 yrs	M/F RATIO: 1/3.4	22.7%	14%	32%	9%	32%
Chiloiro et al. [86]	PAH	21	40 yrs	M: 19% F: 80.9%	33.3%	14.3%	42.8%	23.8%	47.6%
Felix Amereller et al. [87]	PAH	60	N.A.	M: 27% F: 73%	67%	57%	52%	20%	N.A.
Honegger et al. [88]	PAH	76	41 yrs	M: 29% F 71%	47%	48%	62%	37%	54%
Imber et al. [82]	PAH	21	37.4 yrs	M: 38% F: 62%	57%	57%	71.4%	61.9%	47.6%
Imga et al. [89]	PAH	12	44 yrs (17–61)	M: 25% F 75%	58%	75%	75%	50%	17%
Korkmaz et al. [84]	PAH	17	31	M:41.2% F:58.8%	58.8%	52.9%	47.1%	5.9%	47.1%
Khare et al. [90]	PAH	24	N.A.	M: 12.5% F 87.5%	75%	58.3%	50%	N.A.	16.7%
Krishnappa et al. [91]	PAH	39	34.9	M/F RATIO 1: 4.6	64%	56%	54%	N.A.	18%
Park et al. [81]	PAH	22	48 yrs	M: 22.7% F: 77.3%	36.3%	36.3%	31.8%	22.7%	81.8%
Oguz et al. [92]	PAH	20	41.5 yrs	M: 25% F: 75%	35%	61%	66%	21%	28%
Wang et al. [93]	PAH	50	37.2	M: 34% F: 66%	26%	N.A.	60%	22%	72%
Chiloiro et al. [97]	IIH	9	59 yrs (41–83)	M: 66.7% F: 33.3%	100%	0%	0%	0%	0%
Ryder et al. [98]	IIH	19	N.A.	M: 57.9% F: 42.1%	84.2%	57.9%	38.5%^a^	N.A.	N.A.
Faje et al. [99]	IIH	17	68.2 yrs	M: 88.2% F: 11.8%	41.2%	100%	82.3%	N.A.	N.A.
Albarel et al. [100]	IIH	15	55.5 yrs	M: 66.7% F: 33.3%	73.3%	86.7%	80%	N.A.	N.A.
Le Min et al. [101]	IIH	25	N.A.	M: 76% F: 24%	88%	88%	60%	12%	N.A.
Scott et al. [102]	IIH	11	67.8	M: 65% F: 35%	63.6%	63.6%	63.6%	N.A.	N.A.
Jessel et al. [103]	IIH	69	64 (32–83)	M: 68.1% F: 31.9%	68.1%	34.8%	75%	N.A.	N.A.
Kotwal et al. [104]	IIH	26	57.9	M: 54% F: 46%	100%	N.A.	N.A.	N.A.	N.A.
Hong Chen et al. [105]	IIH	28	61.2	M: 57.1% F: 42.9%	53.6%	46.4%	N.A.	N.A.	N.A.

*N.A.* data not available, *M* male, *F* female, *ACTH* AdrenoCorticoTropic Hormone, *TSH* Thyroid-Stimulating Hormone, *FSH* Follicle-Stimulating Hormone, *LH* Luteinizing Hormone, *Yrs* years, *AVP* Arginine VasoPressin, *GH* Growth Hormone

^a^Evaluated in 13 patients

The concomitant occurrence of ICI-induced thyroiditis may interfere with the interpretation of the pituitary secretion test, making the diagnosis of IIH difficult. ACTH deficiency is more common in patients treated with monoclonal antibodies directed against programmed cell death protein 1 (PD-1) and its ligand (PD-1L) than in those treated with monoclonal antibodies directed against cytotoxic T-lymphocyte antigen 4 (CTLA-4) [110]. Recently, the isolated ACTH deficit has been suggested as a clinical manifestation of paraneoplastic syndrome in patients with anti-Pit1 autoantibodies [111], and in the absence of typical radiological signs of hypophysitis. Hypopituitarism with normal radiological findings is described in approximately 77% of reported cases [112]. Taking all these considerations into account, a new classification of ICI-induced pituitary dysfunction has recently been proposed, defining as IIH those cases with new-onset hypopituitarism (with at least two pituitary hormone deficiencies) and identification of typical findings of hypophysitis on radiological studies; as immunotherapy-induced hypopituitarism those cases with new-onset hypopituitarism (with at least two pituitary hormone deficiencies) in the absence of the typical findings of hypophysitis on radiological studies; and as immunotherapy-induced isolated ACTH deficiency those cases with no other pituitary hormone disorder and also in the absence of typical findings of hypophysitis on radiological studies [113].

### Pituitary metastasis

The pituitary gland is a rare but possible site of metastasis. The occurrence of pituitary metastases is associated with a poor prognosis, particularly in patients with additional metastatic sites. The presentation of a pituitary metastasis is variable, with symptoms/signs of hypopituitarism (anterior pituitary dysfunction and/or AVP-D) or symptoms due to compression of adjacent anatomical structures, such as visual field defects and cranial nerve palsies [114]. They may grow rapidly and be locally very aggressive, with the onset of neurological symptoms such as headache, visual and oculomotor disturbances, or endocrinological signs such as AVP-D and hypopituitarism [115].

Differential diagnosis with other sellar lesions is challenging as it may mimic the clinical features of various pituitary disorders. The treatment of pituitary metastases includes surgical decompression, radiotherapy, and chemotherapy. Although earlier literature suggested that the most common sign of pituitary metastasis was AVP-D, more recently an increased incidence of anterior hypopituitarism has been reported, with a prevalence of ACTH deficiency ranging from 25 to 67% of reported case series and TSH deficiency ranging from 27 to 50%, as shown in Table 7 [114, 116–118].

**Table 7:** Epidemiology of ACTH deficit and other pituitary hormones deficit in sellar and parasellar not secreting pituitary lesions

**Authors (Reference)**	**Etiology**	**Num of patients**	**Age at diagnosis** **mean (ranges)**	**Gender** **M/F (%)**	**Frequency of pituitary hormones deficit**				
					**ACTH-** **deficit**	**TSH-** **deficit**	**FSH/LH-** **deficit**	**GH-** **deficit**	**AVP-** **deficit**
Henry et al. [114]	Metastasis	4	46.5 (19–73)	M: 75% F: 25%	25%	50%	75%	N.A.	25%
Javanbakht et al. [116]	Metastasis	11	59.2 (43–76)	M: 54.5% F: 45.5%	27.3%	27.3%	27.3%	27.3%	27.3%
Lithgow et al. [117]	Metastasis	18	61.5	M: 22.2% F: 77.8%	67%	46%	85%	N.A.	N.A.
Mattogno et al. [118]	Metastasis	2	32	M: 100% F: 0%	50%	N.A.	N.A.	N.A.	N.A.
Partenope et al. [129]	Germinoma	55	11 (8–14)	M: 32.7% F: 67.3%	54.5%	49.1%	49.1%	54.5%	52.7%
Chang et al. [131]	Germinoma	49	13.6 (7.5–19)	M: 75.5% F: 24.5%	42.8%	30.6%	8.1%	36.7%	55.1%
Xiang et al. [134]	Germinoma	77	18	M: 80.5% F: 19.5%	57.3%	56%	56.6%	N.A.	52.1%
Moszczyńska et al. [136]	Germinoma	23	9.68	M: 56.5% F: 43.5%	65.2%	69.6%	47.8%	47.8%	69.6%
Brys et al. [141]	Histiocytosis	3	45.3 (41–51)	M: 75% F: 25%	0%	25%	75%	N.A.	100%
Huo et al. [142]	Histiocytosis	7	28 (9–47)	M: 42.8% F: 57.2%	42,8%	42,8%	57,1%	57,1%	100%
Montefusco et al. [143]	Histiocytosis	18	42	M: 38.9% F: 61.1%	5.5%	16.7%	27.8%	27.8%	5.5%

*N.A.* data not available, *M* male, *F* female, *ACTH* AdrenoCorticoTropic Hormone, *TSH* Thyroid-Stimulating Hormone, *FSH* Follicle-Stimulating Hormone, *LH* Luteinizing Hormone, *Yrs* years, *AVP* Arginine VasoPressin, *GH* Growth Hormone

Henry et al. reported four patients with large B-cell lymphoma, multiple myeloma and two cases of unknown primary cancer [114]. In these cases, AVP-D was the most common pituitary dysfunction (about 50% of cases), while anterior pituitary deficiency was reported in 25–45% of cases, with central hypothyroidism and hypocortisolism being more common. Hyperprolactinemia was also common (affecting 2 out of 3 patients). SAI was diagnosed in one in four patients: the diagnosis was made with ACTH test. Breast cancer and lymphoma were the most common primary causes in the study by Javanbakht et al. In this study, panhypopituitarism (3/11 cases) was the most common clinical manifestation of metastasis, and visual symptoms were also seen in 2 of 11 patients [116]. Watanabe et al. [119] described a patient with metastatic lung adenocarcinoma who developed AVP-D and panhypopituitarism: these symptoms led to the diagnosis of pituitary metastasis. The diagnosis of CAI was made by measuring plasma ACTH and cortisol levels.

According to several case reports we found that vision loss appears to be a common clinical presentation of the disease. In 2017, Wendel et al. [120] reported a case of pituitary metastasis from renal cell carcinoma that presented with progressive deterioration of visual acuity and visual field, and whose laboratory tests showed panhypopituitarism. In 2020, Li et al. [121] reported another case of pituitary metastasis from clear cell renal cell carcinoma in a patient with a history of visual disturbances and headaches, which was difficult to distinguish from other tumors in the sellar region. The endocrinological assessment included the measurement of plasma ACTH, cortisol, TSH, FT3, FT4, GH, PRL, LH, FSH, estradiol, pregnenedione, testosterone. In the case series by Lithgow et al., visual dysfunction was the most common presenting manifestation, occurring in 50% of patients [117]. Gonadotrophin, ACTH and TSH deficiencies were present in 85%, 67% and 46% of cases respectively, while AVP-D was diagnosed in 17% of cases. Adrenal insufficiency was defined as a 9 am serum cortisol < 100 nmol/L in the absence of exogenous corticosteroid therapy or an inadequate response to the 250 mcg ACTH short test. Another cause of pituitary metastases is melanoma [122, 123]. Two cases were described by Mattogno et al. [118]. The first case presented with a 3-month history of headache unresponsive to common analgesics, whereas the second case complained of a 2-month history of diplopia and left-sided visual impairment with partial ptosis. In the first case, there was no deficit in pituitary function, whereas in the second, endocrinological examination revealed mild anterior hypopituitarism requiring hormone replacement therapy. Costanza et al. reported the case of a patient with a negative cancer history who presented with a single pituitary metastasis, which was identified as colorectal cancer by immunohistochemistry after surgery: this tumor was diagnosed based on the manifestations of the metastasis, including mass effect and anterior hypopituitarism [124]. Diagnosis can be challenging as pituitary incidentalomas and benign lesions are also reported in cancer patients. Helton et al. reported an anecdotal clinical case of a patient with clear cell renal carcinoma who was diagnosed with a non-functioning pituitary adenoma after the onset of visual disturbances [125].

### Pituitary germinoma

Germinomas are gonadal neoplasms that rarely occur extra-gonadally in the midline structures of the human body. Newly diagnosed adult cases of pineal gland germinoma are very rare, with most cases being diagnosed in the mid-teens [126], with an incidence ranging from 0.4 to 3.4%. The pituitary region is the second most common site of germ cell neoplasia after the pineal gland. Germinomas are primarily treated with radiotherapy with high rates of cure [127], with the possibility of adjuvant chemotherapy [128]. Otherwise, the primary benefit of surgery is the establishment of the pathological diagnosis and the possibility of achieving debulking of the lesion [127]. Partenope et al. analyzed 55 children diagnosed with intracranial germ cell tumors with a median follow-up of 78.9 months from diagnosis. At the time of tumor diagnosis, 50.9% of patients had endocrinopathies: AVP-D in 85.7%, ACTH deficiency in 57.1%, central hypothyroidism in 50%, GH deficiency in 28.5%, hypogonadotropic hypogonadism in 10.7%, and precocious puberty in 10.7%. In these patients, adrenal insufficiency was diagnosed on the basis of the morning serum cortisol or in response to the low-dose ACTH test or the insulin-induced hypoglycemia test. AVP-D, hypothyroidism and SAI occurred earlier than other abnormalities and often preceded the identification of a pituitary lesion [129]. It is clear from this study that endocrinopathies are common presenting findings of intracranial germ cell tumors and may also develop months to years after cancer diagnosis. AVP-D is often the first sign leading to the diagnosis of the disease [130]. In the study by Chang et al., GHD was the most common pituitary dysfunction, followed by adrenal insufficiency, and almost half of the patients presented with AVP-D [131]. In addition, Zilbermint et al. described a patient with hypopituitarism (central hypothyroidism, hypogonadotropic hypogonadism, adrenal insufficiency, and central diabetes insipidus), with no data available for GHD [132]. Ventura et al. [133] reported a bifocal germinoma leading to ACTH deficiency, diabetes insipidus, hypogonadotropic hypogonadism, and secondary hypothyroidism. Xiang et al. [134] reported that 57.3% of patients had SAI. Tian et al. wrote an interesting case report of a patient with a germinoma diagnosed after finding an undetectable TSH plasmatic levels [135]. Germinomas should be considered in the differential diagnosis of patients with anterior pituitary deficits or AVP-D, as shown by Moszczynska et al., who diagnosed adrenal insufficiency by serum cortisol level at 8 am and ACTH test (1 µg i.v.) [136]. As shown in Table 7, ACTH deficiency is reported in 43% to 57% of cases, TSH deficiency in 31% to 57%, FSH/LH deficiency in 8% to 57%, GH deficiency in 37% to 54% and AVP-D in 51% to 70% of cases [129, 131, 134, 136].

### Langerhans cell histiocytosis

Langerhans cell histiocytosis is a neoplastic infiltrative disorder characterized by the proliferation of abnormal antigen-presenting immune cells called Langerhans cells. The name is derived from the similarity between neoplastic cells and dendritic Langerhans cells of the skin and mucosa. Neoplastic cells are CD1a+ and CD207+ and derive from myeloid dendritic cells. It is usually diagnosed in childhood [137]. Less than 30% of cases are reported in adults, with an incidence of 1.8 per million population [138]. The disease has features of either an abnormal reactive process or a neoplastic process [139]. Langerhans cell histiocytosis affects most commonly bones and skin, but also affect bone marrow, liver, spleen, lymph nodes, pituitary gland, central nervous system, gastrointestinal tract, lungs, and other organs. The diagnosis of this disease is very challenging due to the occurrence of several diseases with similar characteristics and the rarity of this pathology. The prognosis varies depending on the presentation and the degree of systemic and multi-organ involvement. Pituitary infiltration is characterized by progressive pituitary dysfunction and a poor prognosis. Langerhans cell histiocytosis is mostly characterized by AVP-D, but sometimes patients may also present with anterior hypopituitarism [140]. The frequency of central hypoadrenalism in the reported case series ranged from 5 to 100%: this reflects the small number of patients and the variability of presentation, as shown in Table 7 [141–143]. Ryan et al. reported a case of isolated ACTH deficiency in patients with Langerhans cell histiocytosis with normal ADH secretion function [144]. Similarly, Radojkovic et al. described a patient who developed AVP-D and permanent partial anterior hypopituitarism in only 3 months after surgery for Langerhans cell histiocytosis [145]. Huo et al. also showed that all patients with isolated hypothalamic-pituitary Langerhans cell histiocytosis presented with AVP-D as the initial sign, but more than half (4/7) of the patients subsequently developed an anterior pituitary deficit: the diagnosis of CAI was based on ACTH and cortisol levels and, if necessary, an insulin tolerance test [142]. Conversely, Brys et al. [141] and Kadowaki et al. [146] described four patients with partial hypopituitarism but preserved ACTH secretion. Montefusco et al. reported SAI (diagnosed with basal ACTH and cortisol and with ACTH test) in 1/18 patients [143]. Furthermore, this condition is difficult to treat, and the progressive loss of endocrine function is irreversible in most cases. Reviewing data from previous case series, ACTH deficiency occurs in 0 to 5% of cases, TSH deficiency in 16 to 25% of cases, FSH/LH deficiency in 28 to 75% of cases, GHD in approximately 28% of cases, and AVP-D in 5 to 100% of cases ([141–143]).

## Effects of different glucocorticoid therapeutic regimens in SAI

The treatment of patients with SAI must address the suppression of the hypothalamic-pituitary-adrenal axis associated with the disruptive effect of pituitary disease on ACTH secretion. Patients must therefore be treated with glucocorticoid replacement therapy to simulate the physiological circadian rhythm of cortisol secretion. To best manage this condition, it is essential to pay close attention to the dose and timing of glucocorticoid administration [147].

Many studies have shown that patients with SAI on replacement therapy have an increased prevalence of cardiovascular risk factors and adverse metabolic profiles, such as abdominal obesity, elevated blood pressure, dyslipidaemia [148], glucose intolerance or impaired fasting glucose [8, 149]. Exposure to excessive doses of exogenous glucocorticoids has been suggested to play a key role in altering the glucometabolic profile and increasing cardiovascular risk [150]. In a retrospective and observational study of 8818 patients, Stewart and co-authors demonstrated that treatment with hydrocortisone (at a dose of less than 50 mg daily) was associated with an increased risk of arterial hypertension, type 2 diabetes mellitus and dyslipidaemia compared with healthy controls [151].

Data from real-world clinical practice show significant heterogeneity in treatment regimens according to the type, dose, frequency, and timing of glucocorticoid replacement therapy [152]. It is recommended that therapeutic regimens should be adjusted to reduce the daily dose of hydrocortisone or hydrocortisone-equivalents [45]. However, very few randomised clinical trials have investigated the effects of reducing the daily glucocorticoid dose on glucose metabolism and homeostasis in patients with SAI. Furthermore, specific data in this regard are often influenced by many confounding factors, including the presence of comorbidities and other concomitant replacement therapies [153, 154]. Table 8 summarises the results of trials investigating the effects of glucocorticoid replacement therapy on various systemic outcomes, such as glucose, lipid and bone metabolism, cardiovascular function, and mortality [151, 153, 155–160].

**Table 8:** Systemic effects of glucocorticoid replacement therapy in patients with SAI, according to different drugs, treatment doses and therapy duration

**Authors (Reference)**	**Study design**	**Num of patients**	**Dose and types of glucocorticoid replacement therapy**	**Systemic effect**
Stewart et al. [151]	Retrospective observational	8818	HC less than 50 mg/day for 6–12 months	↑ hypertension, type 2 DM, hyperlipidaemia in patients vs health control
Filipsson et al. [153]	Prospective clinical trial	1707	1168 patients treated with HC 487 patients treated with CA 52 patients treated with PD	After adjustment for glucocorticoid dose: - PD treated patients had higher weight/height ratio; - CA treated patients had lower HbA1c levels, at 12 months of treatment No change in BMI, fasting blood glucose (FBG), total cholesterol, HDL, LDL, triglycerides (TG), at 12 months of treatment
Petersons et al. [155]	Clinical trial	17	≤ 20 mg/day HC or equivalent for a median duration of 32 (range 9 –95) months, then increased to 30 mg/day HC or equivalent for 7 days	↓ fasting AIx75 and fasting RHI with higher HC doses → fasting glucose concentration, 2-h glucose concentration and insulin sensivity
Castinetti et al. [156]	Retrospective observational	122	Mean 19.9 mg daily HC or equivalent for a mean follow-up of 10.8 years	High HC doses in patients with dyslipidaemia and high blood pressure
Hayashi et al. [157]	Prospective Clinical trial	29	10, 20 and 30 mg/day HC for 4 weeks of treatment	↑ of adiponectin and HDL-C in HC-dose dependent manner
Werumeus Buning et al. [160]	Clinical trial	47	Group 1 first received a physiological lower dose of HC for 10 weeks (0.2–0.3 mg/kg/d), followed by a physiological higher dose for another 10 weeks (0.4–0.6 mg/kg/d). Group 2 received the two doses in reverse order	↑ systolic and diastolic blood pressure at higher HC dose treatment
Ragnarsson et al. [158]	Retrospective	204	Mean HC or equivalent dose 20,5 ± 5,8 mg/day	↓ BMD at lombar spine and femoral neck and ↑ the frequency of osteopenia in woman
Hammarstrand et al. [159]	Retrospective Swedish national patient register	193	Mean daily HC or equivalent dose of 20 ± 6 mg and HC dose per kg 0.25 ± 0.09	↑ SMR in patients treated with HC or eq dose > 20 mg/day or > 0.3 mg/Kg vs general population → SMR in patients treated with HC or eq dose ≤ 20 mg/day or 0.3 mg/kg/day vs general population

↑ increase, ↓ reduction, → stable, *DM* Diabetes Mellitus, *HC* hydrocortisone, *CA* cortisone acetate, *PD* prednisone, *HCeq* hydrocortisone-equivalents, *FBG* fasting blood glucose, *HbA1c* glycated haemoglobin, *HDL* high-density lipoprotein, *HDL-C* HDL-cholesterol, *LDL* low-density lipoprotein, *TG* triglycerides, *AIx75* augmentation index, *RHI* reactive hyperaemia index, *BMD* Bone Mineral Density, *SMR* standardized mortality ratio

Prednisone treatment was associated with a higher weight/height ratio, whereas cortisone acetate treatment did not appear to be associated with a worsening of glycosylated haemoglobin [153].

In an interventional study of 17 patients with SAI, Petersons et al. evaluated the metabolic and cardiovascular effects of short-term (seven days) therapy with high-dose hydrocortisone or equivalent (30 mg daily) in patients previously treated with less than 20 mg/day hydrocortisone or equivalent [155]. The use of a higher glucocorticoid regimen was not associated with significant differences in fasting or 2-h glucose concentrations or insulin sensitivity. On the other hand, fasting augmentation index and reactive hyperaemia index were lower with higher doses of hydrocortisone or equivalent, suggesting that endothelial dysfunction, probably as a direct glucocorticoid effect, may contribute to the increased cardiovascular mortality associated with over-replacement.

An observational study of 122 patients with SAI by Castinetti et al. showed no significant correlation between hydrocortisone or equivalent dose and body mass index (BMI) or other metabolic parameters, although there is some evidence that overdosing contributes to metabolic impairment. This study showed an association between the use of higher doses of hydrocortisone and the presence of dyslipidaemia and hypertension, although there was only a small dosage difference between patients with and without dyslipidaemia and between patients with and without hypertension [156]. On the other hand, glucocorticoid administration has been shown to increase serum adiponectin and HDL-C levels in a dose-dependent manner in patients treated with hydrocortisone [157].

The effect of relatively high and low glucocorticoid doses on the cardiovascular profile was also investigated in a Dutch clinical trial by Werumeus Buning et al. involving 47 patients with SAI. The cohort was divided into two groups: group 1 received a lower dose of hydrocortisone for 10 weeks (0.2–0.3 mg/kg/d) followed by a higher dose for a further 10 weeks (0.4–0.6 mg/kg/d), while group 2 received these two doses in reverse order. During exposure to the higher dose of hydrocortisone, patients showed a significant increase in both systolic and diastolic blood pressure compared with the values observed when the lower dose was administered to the same patients [160].

Moreover, a higher daily hydrocortisone equivalent dose has been associated with a reduction in quality of life [161, 162]: in particular, Blacha et al. do not report an impact on sleep quality or daytime sleepiness, but they find a negative impact on mental health.

In addition to dosage, another important point of discussion is the time of administration: in patients with SAI, Barlas et al. [163] showed an association between morning hydrocortisone replacement time and the Pittsburgh Sleep Quality Index, whereas no association was found between hydrocortisone replacement therapy and sleep quality.

Regarding bone mass density (BMD), it has been shown [164] that BMD is not lower in patients with hypoadrenalism receiving replacement therapy with a mean hydrocortisone dose of 20 mg daily compared with patients without ACTH deficiency. Studies on PAI have also demonstrated that reducing the hydrocortisone equivalent can improve BMD [165]. However, women with ACTH deficiency have reduced bone mineral density regardless of the daily hydrocortisone equivalent dose [158]. Furthermore, a meta-analysis conducted by Al Nofal et al. [166] demonstrated that patients on conventional steroid treatment exhibited significantly poorer bone parameters, accompanied by an elevated risk of fractures. Hammarstrand et al. evaluated 392 patients and highlighted that a daily hydrocortisone-equivalent dose > 20 mg was associated with higher mortality, whereas patients taking doses ≤ 20 mg had a mortality risk like that of patients without glucocorticoid replacement and the general population [159].

Notable findings supporting the need to avoid overdosing were highlighted in a Swiss study [167] of 105 patients receiving glucocorticoid replacement therapy for SAI after pituitary surgery for non-functioning Pit-NETs. In this study, patients were divided into three groups according to the daily administered dose of hydrocortisone or equivalent: 0.05–0.24 mg/kg/d hydrocortisone or equivalent, 0.25–0.34 hydrocortisone or equivalent, and ≥ 0.35 mg/kg/d hydrocortisone or equivalent. The study provided evidence that a higher dose of glucocorticoid replacement (greater than 0.24 mg/kg/day) was associated with increased mortality, with a hazard ratio of 2.62 for the group of patients treated with 0.25–0.34 mg/kg/day hydrocortisone or equivalent and a hazard ratio of 4.56 for the group of patients treated with ≥ 0.35 mg/kg/day hydrocortisone or equivalent [167].

### Potential advantages of modified release glucocorticoid administration

Increased awareness of the detrimental effects of over-replacement has led to the introduction of new modified release formulations of hydrocortisone, with the aim to provide a cortisol exposure-time profile closer to the physiological profile, as shown in Table 9 [32, 168–171].

**Table 9:** Systemic effects of dual release glucocorticoid replacement therapy in patients with SAI

**Authors (Reference)**	**Study design**	**Number of patients**	**Dose and types of glucocorticoid replacement therapy**	**Systemic effect**
Quinkler et al. [168]	Clinical trial	9	Dual-release HC therapy (median dose 20 mg/d), after 6 months of treatment with conventional HC or eq	↓ BMI and HbA1c in dual reseal HC
Isidori et al. [169]	Clinical trial	46	Dual-release HC therapy for 24 weeks, after 48 months of treatment with conventional HC or eq	↓ bodyweight and better immune cells profiles in patients switched to modified-release oral hydrocortisone
Frara et al. [32]	Observational Retrospective	14	Mean 20 mg daily dual-release hydrocortisone	↓ FBG and → HbA1c, LDL, HDL, TG at 24 months of treatment ↑ BMD T-score at lumbar spine, femoral neck and total hip during 24 months of treatment
Guarnotta et al. [170]	Retrospective observational	36	Mean 20 mg/d dual-release hydrocortisone for 36 months of treatment	↓ BMI, waist circumference and HbA1c ↑HDL-C levels in patients with normal glucose tolerance and pre-diabetes ↓ Insulinemia, visceral adiposity index (VAI), insulin resistance, ↑ insulin sensitivity in prediabetic patients
Dineen et al. [171]	Clinical trial	30	Dual-release hydrocortisone for 3 months of treatment	↓ systolic and diastolic blood pressure, body weight and BMI

↑ increase, ↓ reduction, → stable, *DM* Diabetes Mellitus, *HC* hydrocortisone, *CA* cortisone acetate, *PD* prednisone, *HCeq* hydrocortisone-equivalents, *FBG* fasting blood glucose, *HbA1c* glycated haemoglobin, *HDL* high-density lipoprotein, *HDL-C* HDL-cholesterol, *LDL* low-density lipoprotein, *TG* triglycerides, *AIx75* augmentation index, *RHI* reactive hyperaemia index, *BMI* Body Mass Index, *VAI* visceral adiposity index

Quinkler et al. conducted a prospective study of 18 patients with SAI: 9 patients were switched to modified-release hydrocortisone. This study showed that modified-release hydrocortisone reduced BMI and HbA1c compared with conventional glucocorticoids [168]. The effect of once-daily extended-release hydrocortisone on glucose metabolism and cardiovascular profile compared with standard glucocorticoids was investigated in a clinical trial by Isidori et al. involving 45 patients with SAI. This study showed that switching to a once-daily extended-release hydrocortisone regimen resulted in a more physiological circadian glucocorticoid rhythm, leading to a reduction in body weight and an overall improvement in immune function [169].

A significant improvement in the cardio-metabolic risk profile was also observed in a cohort of 36 patients with SAI who were switched from conventional daily hydrocortisone treatment (with a mean daily dose of 20 mg) to dual-release hydrocortisone 20 mg/day for 36 months [170]. Patients with normal glucose tolerance and with pre-diabetes appeared to benefit from extended-release hydrocortisone in terms of reductions in BMI, waist circumference and HbA1c levels.

Dineen et al. [171] showed that once-daily extended-release hydrocortisone reduced systolic blood pressure by 5.7 mmHg and diastolic blood pressure by 4.5 mmHg. It also reduces body weight and BMI. In addition, the reduction in systolic blood pressure seems to be more important in patients with SAI than in those with PAI. Notable findings on the potential benefits of extended-release hydrocortisone came from an Italian study by Frara et al. involving 14 patients with AI [32]. This paper focused on evaluating the effect of extended-release glucocorticoid administration on BMD at the lumbar spine, femoral neck, and total hip. The effect was measured at baseline and after 24 months of treatment using dual-energy X-ray absorptiometry. The study supported the bone safety of the new modified release formulations as the data showed a significant increase in BMD at both the lumbar spine and femoral neck.

## Conclusion

Managing hormone replacement therapy in SAI is complex and constantly evolving. The optimisation and personalisation of glucocorticoid therapy is essential to minimise the risk of overdose and to prevent life-threatening hypoadrenal crises. However, despite significant advances in clinical management, there are still many areas that require further research and innovation, and developing more accurate methods to monitor and adjust glucocorticoid dosing is one of the actual major challenges. Traditional clinical evaluation using anthropometry and biochemistry needs integrations with new technology including continuous monitoring devices and advanced data analysis. These tools could allow more timely and precise adjustment of therapy by providing a more detailed view of hormonal fluctuations and individual metabolic responses.

To better understand the associated risks on the long-term effects of glucocorticorticoid therapy on glucose metabolism, lipid metabolism, bone and cardiovascular function, more effective prevention strategies should be developed. For example, a standard component of SAI management could be the integration of bone mineral density and cardiovascular health surveillance programs.

The use of innovative biomarkers to predict individual response to therapy and to detect early signs of overdose or ineffectiveness is another promising area. Researchs into genetic and molecular markers could open new avenues for personalising therapy, improving clinical outcomes and reducing side effects.

## Data Availability

No datasets were generated or analysed during the current study.
